# Association between intraoperative hypothermia and postoperative delirium: a preliminary meta-analysis

**DOI:** 10.1186/s13643-024-02669-z

**Published:** 2024-09-30

**Authors:** I-Wen Chen, Wei-Ting Wang, Kuo-Chuan Hung

**Affiliations:** 1 Department of Anesthesiology, Chi Mei Medical Center, Liouying, Tainan City, Taiwan; 2 Department of Anesthesiology, E-Da Hospital, I-Shou University, Kaohsiung City, Taiwan; 3 Department of Anesthesiology, Chi Mei Medical Center, No.901, ChungHwa Road, YungKung Dist, Tainan, 71004 Taiwan

**Keywords:** Hypothermia, postoperative delirium, meta-analysis

## Dear Editor,

Intraoperative hypothermia (IOH), defined as a core body temperature below 36 °C, can occur during surgery due to anesthesia-induced impairment of thermoregulation, exposure to a cold operating room environment, and the use of cold intravenous fluids. The occurrence of IOH has recently been identified as a potential risk factor for postoperative delirium (POD) in patients undergoing non-cardiac surgery [1, 2]. Because IOH is not uncommon, clarifying the relationship between IOH and POD and providing a comprehensive analysis of the available evidence are required. Accordingly, we conducted a preliminary meta-analysis of studies that examined the impact of IOH on POD. This meta-analysis aimed to synthesize the current knowledge, identify potential gaps in the literature, and provide insights for future research and clinical practice.

A literature search was conducted using Medline, Embase, Cochrane Library, and Google Scholar from inception to July 2024. The search terms included “hypothermia," "intraoperative," "postoperative cognitive impairment," "postoperative cognitive dysfunction," "postoperative delirium," “neurocognitive recovery," and their synonyms. The search was limited to human studies without language limitations. The reference lists of the included studies and pertinent review articles were manually reviewed to identify other potentially eligible studies. Studies were included if they met the following criteria: (1) observational or interventional studies comparing the incidence of POD between patients with and without IOH. The definition of IOH was based on individual studies, (2) adult patients (aged ≥ 18 years) undergoing non-cardiac surgery, and (3) reporting of sufficient data to calculate effect sizes. Two reviewers independently reviewed the titles and abstracts of the retrieved studies, and full-text articles were acquired for studies that appeared to be potentially eligible.

The primary outcome was POD incidence. The same reviewers independently extracted data from the included studies using a standardized form. The extracted data included study characteristics (e.g., sample size), patient characteristics (e.g., age), methods for POD measurement, and country. The methodological quality of the included studies was assessed using the Newcastle-Ottawa Scale (NOS). Studies with seven or more stars were considered to have a low risk of bias. Effect sizes were expressed as odds ratios (ORs) with 95% confidence intervals (CIs). The combined effect size was estimated using a random-effects model to account for the expected variability among the studies. Heterogeneity was evaluated using the I^2^ statistic, in which values of 75% were interpreted as high levels of heterogeneity. Subgroup analyses were conducted based on the patient population (i.e., age ≥ 60 years vs. age ≥ 18 years). Publication bias was visually assessed using funnel plots. All statistical analyses were performed using the Cochrane Review Manager (RevMan 5.3; Copenhagen: The Nordic Cochrane Center, The Cochrane Collaboration, 2014).

The initial literature search yielded 702 records, of which five studies [1–5] met the inclusion criteria and were included in the meta-analysis. The characteristics of the included studies are summarized in Table 1. All studies were observational and were published between 2018 and 2024. The study was conducted in various countries including Korea (*n* = 3), Japan (*n* = 1), and China (*n* = 1). The sample sizes ranged from 208 to 27,674 patients, and a total of 29156 patients were included in the meta-analysis. Four studies focused on elderly participants (i.e., age ≥ 60 years) [2–5], whereas one study included patients aged ≥ 18 years [1]. Three different tools were used across studies to diagnose POD. The Diagnostic and Statistical Manual of Mental Disorders, fifth edition (DSM-V) was used in two studies [3, 5]. The Confusion Assessment Method (CAM) was employed in two other studies [2, 4]. One study [1] used a chart-based method. NOS scores ranged from 7 to 9, indicating moderate to high quality of the included observational studies.

**Table 1:** Characteristics of five studies

Studies	Population (age; years)	n	Type of surgery	Tool for delirium diagnosis	Country	NOS
Choi 2020	≥ 65 yr	446	Spinal fusion surgery	DSM-V	Korea	9
Hiraki 2021	≥ 70	208	Colorectal cancer surgery	Confusion Assessment Method	Japan	9
Ju 2023	≥ 18	27,674	Non-cardiac surgery	Chart-based method	Korea	7
Kim 2018	≥ 65	318	Total knee arthroplasty	DSM-V	Korea	8
Wang 2024	64–68	510	na	Confusion Assessment Method	China	8

*DSM-V* Diagnostic and Statistical Manual of Mental Disorders, fifth edition, *NOS* Newcastle-Ottawa Scale, *na* not available

**Fig. 1:**
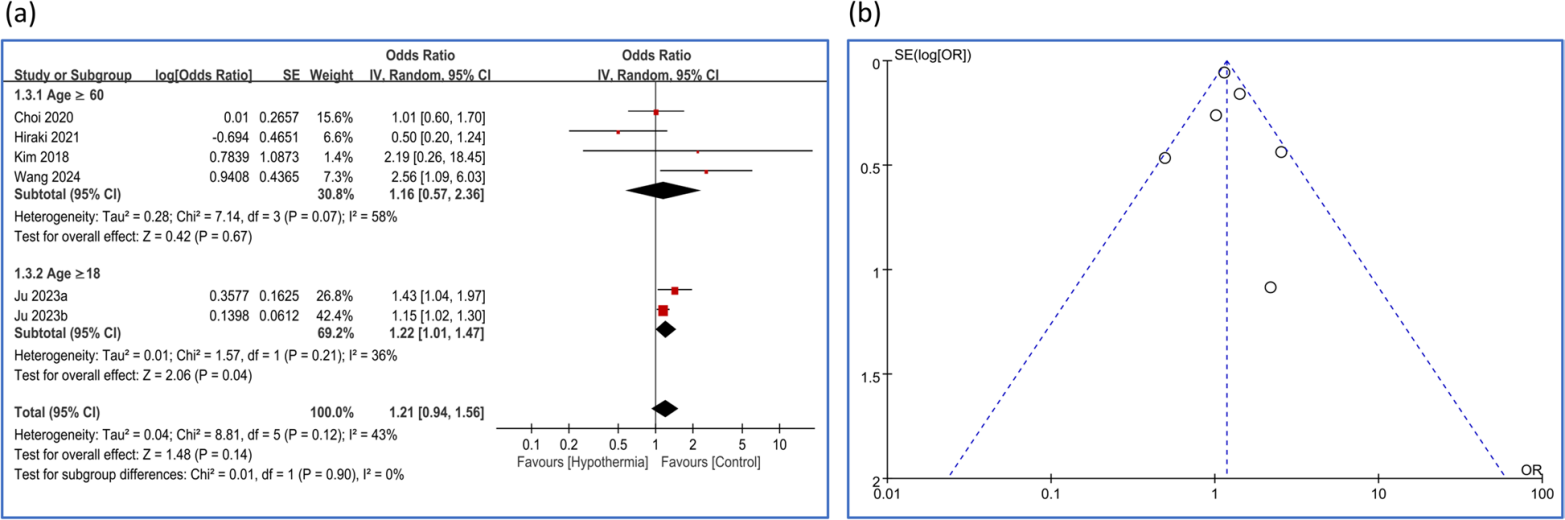
Forest plot and funnel plot of the association between intraoperative hypothermia and postoperative delirium (POD) risk. **a** Forest plot presenting odds ratios (ORs) and 95% confidence intervals (CIs) for the association between intraoperative hypothermia and POD risk. Studies are grouped by age: ≥ 60 years and ≥ 18 years. **b** Funnel plot assessing potential publication bias

The pooled results and subgroup analysis are shown in Fig. 1. One study by Ju et al. [1] provided two datasets examining the association between POD and different levels of hypothermia: mild hypothermia (35.0 °C to 36.0 °C) and severe hypothermia (below 35.0 °C). These datasets were included in the analysis. Overall, there was no significant association between IOH and POD (OR:1.21, 95% CI: 0.94-1.56, *p* = 0.14). In the subgroup of patients aged 60 and over, the pooled OR was 1.16 (95% CI: 0.57-2.36, *p* = 0.67), indicating no significant association between IOH and POD (Fig. 1a). For the subgroup aged 18 and over, the pooled OR was 1.22 (95% CI: 1.01-1.47, *p* = 0.04), suggesting a significant 22% increased risk of POD associated with IOH. A funnel plot (Fig. 1b) was used to visually assess potential publication bias. The plot appeared symmetrical, suggesting no clear indication of publication bias.

The current meta-analysis found no significant association between IOH and the risk of POD in non-cardiac surgery, consistent with the results reported in cardiac surgery [6]. Subgroup analysis suggested that the impact of IOH on POD risk may vary by age. The lack of a significant association in the older subgroup could be attributed to several factors. First, older patients may have a higher baseline risk of POD owing to age-related changes in brain function, comorbidities, and polypharmacy [7]. This higher baseline risk might make it more difficult to detect incremental effects of IOH. Second, studies in the older subgroup had smaller sample sizes and wider confidence intervals, which could have limited their statistical power to detect a significant association. This meta-analysis had several limitations. First, the included studies were observational in nature, which may introduce potential confounding factors and bias. Second, the definition and assessment of POD varied among the studies, leading to heterogeneity in the results. Third, most studies were conducted in Asian populations, which may not be representative of other ethnic groups.

In conclusion, this preliminary meta-analysis suggests that IOH is not associated with increased POD risk. Given the limitations of the included studies, further high-quality research is warranted to establish the relationship between IOH and postoperative cognitive impairment.

## Data Availability

The datasets used and/or analyzed in the current study are available from the corresponding author upon reasonable request.
